# Toxicity to intravitreal melphalan in a patient with retinoblastoma

**DOI:** 10.22336/rjo.2023.49

**Published:** 2023

**Authors:** Marta Gema Solaz-Ruiz, Lorena Azorín Pérez, Carlos Cauto-Picazo, Honorio Barranco-González, Isabel Pascual-Camps, Enrique España-Gregori

**Affiliations:** * La Fe University and Polytechnic Hospital, Valencia, Spain

**Keywords:** intravitreal melphalan, ocular toxicity, phthisis bulbi, hypotonia, retinoblastoma

## Abstract

**Objective:** Description of melphalan’s toxicity in retinoblastoma treatment.

**Methods:** Clinical case report.

**Results:** We presented a case of unilateral retinoblastoma with vitreous seeding at diagnosis, in which the use of intravitreal melphalan produced many adverse reactions.

**Conclusions:** Vitreous seedings have been one of the most important challenges in retinoblastoma treatment. Intravitreal melphalan has achieved the regression of vitreous seedings in a large percentage of cases. It is a safe treatment; however, it can produce toxicity, even with the standard dose of 20-30 µg, which has been poorly documented. Exhaustive follow-up of patients is recommended for an early diagnosis of possible adverse effects.

**Abbreviations: **OS = left eye, RI = magnetic resonance imaging, OCT = optical coherence tomography

## Introduction

Retinoblastoma represents the most prevalent intraocular tumor in the pediatric population [**1**]. It currently benefits from multidisciplinary management, with different treatment modalities, the last available resource being the enucleation [**2**]. 

The use of intravitreal chemotherapy, specifically melphalan, seems to be a promising treatment aimed at the reactivation or persistence of the retinoblastoma’s vitreous seedings, causing the regression of these in up to 87% of cases [**3**]. In 2014, Shields CL et al. proved that the administration of intravitreal melphalan at a dose ranging between 20-30 µg, produced complete regression of vitreous seedings in the total of treated eyes [**4**]. Despite its benefits, melphalan has adverse effects. It has been described that the use of melphalan with doses less than or equal to 30 µg produced a minimal risk of intraocular toxicity in the Caucasian population of Europe and America [**5**]. However, a recent study published in 2019 on the Chinese population, highlighted a higher rate of associated toxicity [**6**]. In addition, Francis et al. described an increase in melphalan toxicity in eyes with higher pigmentation, suggesting that they absorb a higher level of chemotherapy [**7**]. 

## Materials and methods

Clinical case report and revision of the current literature.

## Results

**Fig. 1:**
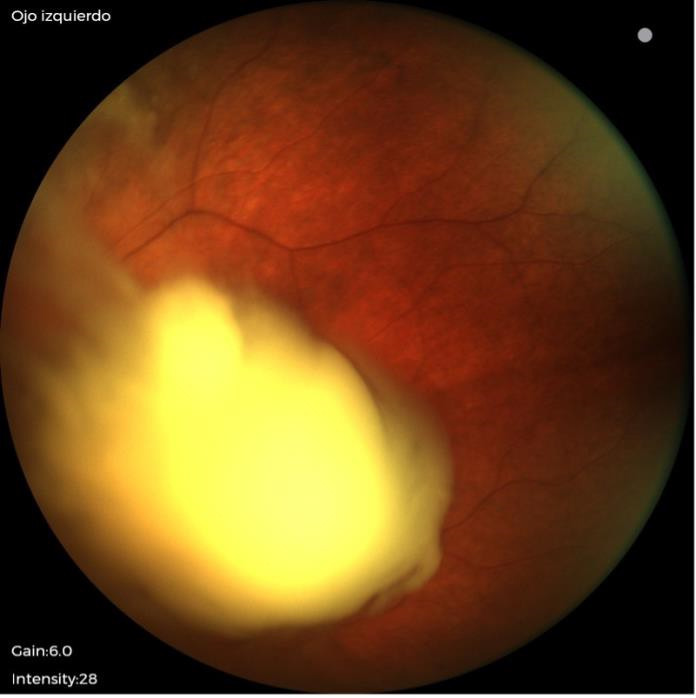
OS fundus. White mass located in the nasal retinal region

A 3-year-old child was diagnosed with unilateral retinoblastoma in his left eye (OS) and vitreous seedings at diagnosis. He was referred to our hospital for leukocoria in his OS. The funduscopic exam showed a white mass located in the nasal retinal region, associating a mobile whitish mass in the vitreous that suggested retinoblastoma (**Fig. 1**). Orbit ultrasound and magnetic resonance imaging (MRI) of the brain were carried out to confirm the diagnosis and determine its dimensions. A genetic study of the RB1 gene did not show the presence of any pathological mutation.

The initial treatment was intraarterial melphalan and topotecan. After that, a partial regression of the mass was observed, with the persistence of type III vitreous seedings (cloud seedings). Subsequently, intravitreal topotecan was administrated at a dose of 20 µg, persisting vitreous seedings after two cycles of treatment, separated two weeks from each other. Afterward, intravitreal melphalan was used on 4 different occasions, observing the decrease of the mass, even though vitreous seedings continued to present. In all intravitreal administrations, the anti-reflux safety protocol was followed correctly.

**Fig. 2:**
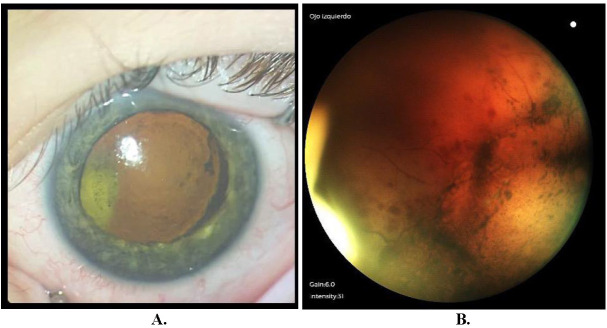
**A** Change of iris coloration towards gray color, pigment deposits in anterior crystalloids, and presence of a bulging lens that impressed subluxate. **B.** OS fundus. Salt and pepper retinopathy, retinal hemorrhages, and persistence of vitreous seedings

**Fig. 3:**
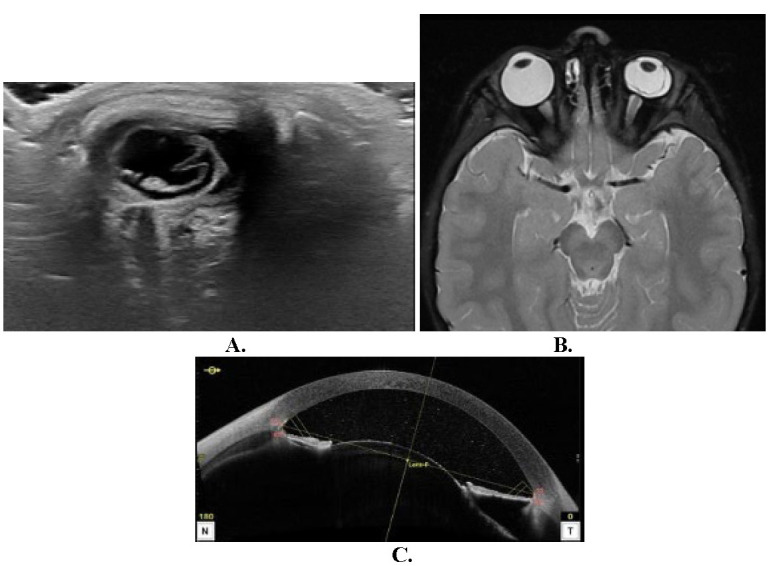
**A** Orbit ultrasound showing phthisis bulbi and choroid detachment. **B.** Brain MRI showing phthisis bulbi choroid detachment. **C.** Anterior OCT revealing choroid detachment

After the last intravitreal melphalan injection, several adverse effects were observed, including a change of iris coloration toward gray color, pigment deposits in anterior crystalloids, and the presence of a bulging lens that impressed subluxate (**Fig. 2A**). Intraocular pressure measured with Perkins’s tonometer was 2 mmHg. Funduscopic examination revealed salt and pepper retinopathy, retinal hemorrhages, and persistence of vitreous seedings (**Fig. 2B**). Orbit ultrasound and brain MRI revealed phthisis bulbi, observed as a loss of eyeball sphericity and a linear image that suggested choroid detachment (**Fig. 3A**, **B**). Anterior optical coherence tomography (OCT) also evidenced these findings (**Fig. 3C**).

he case was presented in a multidisciplinary ophthalmology, radiology, and pediatric oncology committee. Finally, enucleation of the OS was carried out due to the poor visual prognosis, the lack of viability of the eye, and the persistence of the tumor. Post-enucleation histological study revealed no infiltration of the optic nerve, choroid nerve, or anterior chamber. Brain MRI showed no complications and no intraorbital or extraorbital infiltrative neoplastic pathology.

## Discussion

Intravenous chemotherapy and intraarterial chemotherapy are the most common conserving therapies in cases of retinoblastoma. Intravitreal melphalan has emerged as an especially useful therapy for vitreous seedings [**6**]. Intravitreal administration was excluded for decades in the treatment of active retinoblastomas, given the potential risk of tumor spread through the injection site. Subsequently, an anti-reflux safety protocol was developed, which included the administration of the drug in the pars plana, in a tumor-free quadrant, controlling intraocular pressure and freezing immediately the injection path through cryotherapy, avoiding the development of metastases, and allowing intravitreal chemotherapy to be included in the retinoblastoma’s treatment [**3**].

Intravitreal melphalan response is related to the subtype of vitreous seedings. It has been described as an average of 3 injections in type I seedings (powder), 6 in type II (spheres), and 9 in type III (clouds) for their complete regression [**8**]. This phenomenon has been explained by the aggregation of tumor cells in cloud-like seedings, which makes intravitreal chemotherapy difficult to meet the cells in the center of the cloud [**9**]. 

Toxicity related to intravitreal melphalan administration continues to be unclear. There are studies, which proposed that the risk of ocular toxicity decreases with doses below 30 μg [**5**]. For example, Munier and Shields observed complete regressions of vitreous seedings in most cases using these doses, without observing significant adverse effects. However, in recent publications focusing on the Asian population [**6**], a greater number of complications have been documented. These complications could be due, in part, to the fact that this population has mostly pigmented eyes, a fact that has been associated with more toxicity [**7**].

Different forms of toxicity related to the posterior pole associated with the use of intravitreal melphalan have been described, such as salt and pepper retinopathy, which appears in 43-50% of cases [**3**]. Retinal hemorrhages have also been documented in a smaller percentage [**9**]. 

There are few studies describing anterior pole toxicity related to the use of intravitreal melphalan. They include traumatic cataract, depigmentation and thinning of the iris, pupillary synechiae, hypotonia, phthisis bulbi, and scleromalacia [**10**]. The opacity of the lens could be due to the trauma generated by the injection. Regarding depigmentation and thinning of the iris, it is thought that melphalan escapes to the anterior chamber damaging the iris stroma. Hypotonia seems to be an extension of the retinal necrosis, choroidal atrophy, and retinal pigment epithelium involvement and is related to a high concentration of drugs at the injection site. Scleral thinning could appear due to the ocular surface’s atrophy or necrosis [**10**]. 

## Conclusions

Intravitreal melphalan is a promising treatment in retinoblastoma management, aimed especially at the persistence or reactivation of vitreous seedings. It is a safe treatment; however, it can produce toxicity, even with the standard dose of 20-30 μg.

Exhaustive follow-up of patients is recommended for an early diagnosis of possible adverse effects.

**Conflict of Interest statement**

The authors declare no conflict of interest.

**Informed Consent and Human and Animal Rights statement**

Informed consent has been obtained from all individuals included in this study. Minor patient consent to publish the case and images were gathered.

**Authorization for the use of human subjects**

Ethical approval: The research related to human use complies with all the relevant national regulations, and institutional policies, is by the tenets of the Helsinki Declaration, and has been approved by the Ethical Committee of La Fe University and Polytechnic Hospital, Valencia, Spain.

**Acknowledgments**

None.

**Sources of Funding**

The authors received no financial support for the research, authorship, and/or publication of this article.

**Disclosures**

None.
